# Experimental Analysis of Combustion and Emission Characteristics of a Diesel Engine Using Diesel‐Biodiesel‐Methanol Blends With Cetane Enhancer Additive Under Pilot Injection Mode

**DOI:** 10.1002/gch2.202500546

**Published:** 2026-03-05

**Authors:** Hiren Dave, Himanshu Choksi, Choon Kit Chan, Mohd Aamir Mumtaz, Nithesh Naik

**Affiliations:** ^1^ Mechanical Engineering Department Pandit Deendayal Energy University Gandhinagar India; ^2^ Chemical Engineering Department Pandit Deendayal Energy University Gandhinagar India; ^3^ Faculty of Engineering and Quantity Surveying INTI International University Nilai Negeri Sembilan 71800 Malaysia; ^4^ Civil Engineering Department Imam Mohammad Ibn Saud Islamic University (IMSIU) Riyadh Saudi Arabia; ^5^ Manipal Institute of Technology Manipal Academy of Higher Education Manipal India

**Keywords:** biodiesel, cetane enhancer, diesel engine combustion, emissions, pilot injection, process innovation

## Abstract

The presented work explores diesel pilot injection (PI) mode with a prime objective of resolving the inherent problem of increased smoke emissions associated with it. The problem of greater smoke emissions under PI mode was addressed by using methanol and a cetane enhancer (CE) with the base fuel. PI mode was investigated by varying pilot injection timing (PIT) as well as pilot injection ratio (PIR) using B30 (30% Biodiesel + 70% Diesel) as a base fuel and single injection (SI) mode with the base fuel served as a reference condition. The biodiesel for the presented study was derived from the waste cooking oil using standard transesterfication process. Methanol was added by 10% volume into B30 to prepare M10 blend and A1 blend was prepared by adding 0.5% volume of di‐tert‐butyl peroxide (DTBP) which is a CE into M10 blend. Experiments were performed using an automotive diesel engine which was operated at rated torque speed (1600 rpm) and 90% of full load conditions. The results demonstrated that A1 blend with 10% PIR and −30° after top dead center PIT improved fuel consumption and thermal efficiency by 8.77% and 13.77% respectively compared to reference condition along with offering reduction in carbon monoxide (CO), nitrogen oxides (NO_x_) and hydrocarbons (HC) emissions by 66.66%, 6.43%, and 8.11% respectively while increasing smoke emissions just by 2.65%.

## Graphical abstract

The work represented in the manuscript is based on resolving the major issue of increased smoke emissions under pilot injection mode while maintaining the core benfits of it, that is, lower nitrogen oxides (NO_x_) emissions and improved fuel economy. This was achieved by inclusion of methanol and cetane enhancer (CE) in the base fuel.

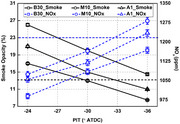

**Abbreviations**
ATDC: After Top Dead Center
BD: Biodiesel
BP: Brake Power
BSFC: Brake Specific Fuel Consumption
BTE: Brake Thermal Efficiency
CA: Crank Angle
CC: Combustion Chamber
CD: Combustion Duration
CE: Cetane Enhancer
CGP: Cylinder Gas Pressure
CN: Cetane Number
CO: Carbon Monoxide
CV: Calorific Value
DP: Delay Period
DTBP: Di‐Tert‐Butyl Peroxide
EGT: Exhaust Gas Temperature
EHN: Ethylhexyl Nitrate
EOC: End of Combustion
HC: Hydrocarbons
HRR: Heat Release Rate
HTA: High Temperature Atmosphere
IP: Indicated Power
MPRR: Maximum Pressure Rise Rate
ND: Neat Diesel
NO: Nitric Oxide
NO_x_
: Nitrogen Oxides
ON: Octane Number
PI: Pilot Injection
PIR: Pilot Injection Ratio
PIT: Pilot Injection Timing
PM: Particulate Matter
SI: Single Injection
SIT: Self‐ignition Temperature
SOC: Start of Combustion
TDC: Top Dead Center
WCO: Waste Cooking Oil
B30: 70% Neat Diesel + 30% Biodiesel
M10: 90% B30 + 10% Methanol
A1: 89.5% B30 + 10% Methanol + 0.5% DTBP

## 1 Introduction

Diesel engines hold a dominating position in sectors like transportation, power generation, earth‐moving equipments, agriculture as well as marine application and it is difficult to challenge their role in these sectors. Considering this, it is of prime of importance to explore alternatives of fossil diesel fuel, which are sustainable and less harmful to the environment in order to reduce dependency on crude oil as well as to meet the ever rising energy demands [1]. At the same time, it also important to control major emissions like nitrogen oxides (NO_x_) and soot from diesel engines considering their adverse impacts on human health and the atmosphere [2].

Biodiesel (BD) is a promising alternate fuel for diesel engines as its cetane number (CN) is close to neat diesel (ND) fuel. In addition to being renewable in nature, it offers several other advantages over fossil diesel fuel like higher lubricity, lower sulfur content, lesser toxicity and reduced carbon based emissions due to fuel bound oxygen [3]. However, higher viscosity, oxidation instability, poor atomization, inferior cold flow characteristics and higher NO_x_ emissions are some major hurdles for wide spread applications of BD [4, 5]. Considering these shortcomings of BD, it is difficult to operate diesel engine using pure BD (B100). However, ND can be partly replaced with BD by blending it with BD in different proportions to get advantage of favorable properties of BD. Many earlier studies reported reduced carbon monoxide (CO), soot/smoke and hydrocarbons (HC) emissions from diesel engines when BD is blended with ND fuel while increasing brake specific fuel consumption (BSFC) and NO_x_ emissions compared to ND operation [6, 7, 8].

Alcohol fuels are another category of oxygenated fuels and have been utilized in diesel engines to control soot emissions [9]. Alcohol fuels are rich in oxygen content and have high octane number (ON) which elongates delay period (DP) of fuel. The longer DP and oxygen content associated with alcohol fuels leads to lower soot formation and better soot oxidation respectively [10]. Among various alcohol fuels, methanol has effectively attracted attention of researchers because of its least carbon content and highest oxygen content (50%) compared to other alcohol fuels [11] due to which it can offer highest reduction in carbon based emissions from diesel engines. However, calorific value (CV) of methanol is considerably lower than petroleum based fuels and reduces engine power considerably when blended with diesel engine fuels [12]. There are three ways by which methanol can be used in diesel engine which include direct mixing [13], dual‐fuel mode operation [14], and direct injection using a separate injector for methanol fuel [15]. The latter two methods are costly and requires major modifications in the existing diesel engines and direct mixing is believed to be the fair and cost‐effective method for utilizing methanol in diesel engines. However, methanol is not miscible with ND fuel due to large difference in polarity between these two fuels and when blended, immediately results in phase separation [16, 17].

There are certain ways proposed in literature [18] to mix methanol with ND, which include usage of emulsifiers and co‐solvents for mixing these two fuels. However, these processes are complex and not economical also [19]. Although methanol is not miscible into ND directly, it is readily miscible into BD [20]. There are many studies which explored diesel engine combustion using methanol with ND using co‐solvents/emulsifiers as well as ND/BD blends using direct mixing. Chen et al. [21] examined diesel engine characteristics by blending methanol with ND for which n‐pentanol was used as co‐solvent. Authors reported that methanol is totally immiscible in ND without addition of n‐pentanol. Increasing methanol quantity in fuel blend caused increased peak heat release rate (HRR), peak cylinder gas pressure (CGP) and peak cylinder temperature. Increased peak cylinder temperature due to methanol addition caused NO_x_ emission to rise while soot emissions were found to be reduced. Zhang et al. [11] assessed diesel engine performance using ND/methanol blends where n‐butanol was used as co‐solvent for mixing diesel and methanol. Authors stated that adding alcohol fuels (methanol and n‐butanol) into ND caused micro‐explosion phenomenon and improved combustion process resulting in lower CO and soot emissions. However, brake thermal efficiency (BTE) and BSFC were deteriorated compared to ND fuel case due to lower CV of alcohol fuels. Also, NO_x_ emissions were reported to be lower for alcohol/ND blends than ND fuel case due to higher latent heat of vaporization of alcohol fuels, specifically methanol. Li et al. [22] examined diesel engine performance using ND/BD/methanol blends. According to the authors, miscibility of methanol into ND improves when BD is used with ND. Qi et al. [23] used B50 (50% ND + 50% BD) as base fuel and explored combustion characteristics using ND/BD/methanol blends where methanol was added in 5% and 10% volumes and blends were denoted as BDM5 (95% B50 + 5% methanol) and BDM10 (90% B50 + 10% methanol) respectively. The study indicated that torque and power output drops slightly due to addition of methanol while soot emissions reduce dramatically. Methanol addition also led to minor reduction in CO emissions while having no significant effect on NO_x_ and HC emissions. Verma et al. [13] assessed diesel engine performance using ND/BD/methanol blends. Authors stated that peak pressure rise rate, peak CGP as well as peak HRR increased when methanol is added as well as it's concentration into blend increases indicating improved combustion process. Methanol addition also led to lower exhaust gas temperature (EGT), shorter combustion duration (CD) along with reduced CO and particulate emissions. However, NO_x_ emissions as well as BTE and BSFC were degraded due to the addition of methanol. Similar results were proposed by several other studies based on ND/methanol or ND/BD/methanol blends [10, 24, 25, 26].

Pilot injection (PI) is a promising technique to lower NO_x_ emissions where a small mass of fuel (called pilot fuel) is injected before the main injection burning of which creates a high temperature atmosphere (HTA) inside combustion chamber (CC). This HTA reduces DP of upcoming main fuel and severity of premixed combustion phase reduces which inhibits NO_x_ formation [27]. Hiren et al. [28] investigated PI mode by varying pilot injection ratio (PIR) and injection pressure. The study reported that PI mode results in advanced combustion phasing compared to single injection (SI) mode and ideal diesel cycle was approcahed more closely. PI mode with 30% PIR offered reduction in NO_x_ as well as BSFC by 21.87% and 1.46%, respectively while increasing smoke opacity by 77.46%. Lu et al. [29] utilized PI mode to improve cold start performance of diesel engine and concluded that PI mode significantly enhances combustion environment in CC and improves cold start performance along with offering NO_x_ reduction. Karthikeyan et al. [30] studied diesel engine performance using BD/methyl acetate blend. According to authors, PI mode reduced BSFC as much as 21.87% compared to SI mode along with offering maximum reduction in HC, NO_x_ and CO emissions by 47.69%, 11.34%, and 56.41%, respectively. Similar results were reported by [31, 32, 33, 34]. Although PI mode offers a noteworthy reduction in NO_x_ emissions, it also increases smoke/soot emissions drastically due to shortening of main fuel DP resulting in lesser time for fuel and air to mix with each other causing increased heterogeneity of the mixture [35]. This is a major drawback of PI mode and many studies have reported greater smoke/soot emissions under PI mode compared to conventional SI mode [31, 32, 33, 34].

Another approach to enhance performance and emission parameters of diesel engines is to modify fuel properties by adding suitable additives. One such important property is cetane number (CN), which governs the DP of fuel and hence affect the overall combustion process and emissions from diesel engines. The most common additives to increase CN of diesel engine fuels (ND/BD/blend of two) are 2‐ethylhexyl nitrate (EHN) and di‐tert‐butyl peroxide (DTBP) as reported in literature. Numerous studies investigated diesel engine outcomes using these two cetane enhancers. Ors [36] operated diesel engine under varying load and constant speed (1400 rpm) condition using ND/BD/ethanol blends while using DTBP as cetane enhancer (CE) in proportions of 1%, 2%, and 3% by volume respectively. Addition of CE offered reduction in CO, smoke and HC emissions up to 22.5%, 24.44%, and 17.44% respectively while increasing NO_x_ emissions and EGT up to 19.55% and 15.22% respectively as reported by the author. Imdadul et al. [37] evaluated diesel engine characteristics using ND/BD/n‐butanol blends along with 2‐EHN as CE. Authors found that addition of 2‐EHN reduced smoke, nitric oxide (NO) and BSFC as much as 14.11%, 5.27%, and 2.8% respectively while increasing HC and CO emissions up to 27.46% and 16.64% respectively. Erol Illeri [38] tested diesel engine fueled with ND/BD/n‐pentanol and ND/BD/n‐butanol blends at constant speed of 2200 rpm and varying load condition while using 2‐EHN as CE. Authors claimed maximum reduction in BSFC and NO_x_ emissions by 8.17% and 5.26% respectively due to the addition of CE. However, CO emissions rose in the range of 7.16%–23.46%. Mishra et al. [39] investigated diesel engine characteristics using ND/BD/ethanol blends in which DTBP was added by 1% on volume. Authors found that combination ND/BD/ethanol/DTBP blends can reduce smoke, HC, NO_x_, and CO emissions up to 45%, 43%, 13%, and 30% respectively compared to the ND case along with improving engine efficiency by 10%. Almost similar results were reported by some other studies also [40, 41, 42].

The following major outcomes can be summarized from the above literature survey:

- Among various alcohol‐based fuels, methanol can offer the highest reduction in soot/smoke emissions from diesel engines as it contains least number of carbons and highest oxygen content.
- Among three available alternatives to use methanol as a fuel in the diesel engines, direct blending of methanol with diesel engine fuels is the most cost‐effective method and can be readily used in existing diesel engines without any modification.
- Methanol and ND are not miscible with each other due to larger difference in polarity between these two fuels. There are two ways by which mixing of these two fuels can be improved. The first one is to use co‐solvent which are mostly long chain hydrocarbon alcohol fuels like butanol, pentanol etc. and the second one is to use some amount of BD with ND.
- Using long chain hydrocarbon alcohol as co‐solvent for mixing methanol with ND increases the number of carbon molecules in alcohol/ND blend and partly compensates the advantage of reduced soot/smoke emissions (due to a greater number of carbon atoms and lesser oxygen content of co‐solvent alcohols compared to methanol) which is the main motive to use alcohol fuels in diesel engines. Therefore, adding BD into ND is a more favorable method in order to obtain maximum soot reduction from the diesel engines, serving the core purpose of using alcohol fuels in diesel engines. In fact, BD addition into ND for improving miscibility of methanol will effectively enhance the soot reduction for ND/methanol blend due to oxygen content of BD.
- PI is an effective technique for controlling NO_x_ emissions from diesel engines as well as to improve its performance. However, it increases soot/smoke emissions drastically which is a major drawback of this technique. Therefore, some techniques are required that can reduce soot emissions under PI mode or at least maintain them at the level of SI mode while maintaining other advantages (like improved engine performance and NO_x_ emissions) of PI mode.
- The addition of CE into diesel engine fuels improves overall performance of diesel engine along with reduction in NO_x_ emissions. However, soot/smoke emissions increase due to the addition of CE.

From the above literature survey, it can be pointed out that many studies have investigated diesel engine's performance using ND/methanol or ND/BD/methanol blends under conventional single injection (SI) mode. However, such studies are very rare under PI mode. There are several studies that explored PI mode in a diesel engine using higher alcohol fuels like n‐butanol [43], n‐pentanol [31] blended with ND or ND/BD blends for controlling soot emissions. However, there are no studies reported based on investigation of PI mode in a diesel engine using methanol as an alcohol fuel directly blended with ND or ND/BD blends although methanol can offer highest reduction in soot emissions due to its lowest carbon content as well as highest oxygen content among all the alcohol fuels as per author's knowledge pointing out a significant research gap. Considering this research gap, the present work is aimed to investigate the diesel engine's combustion, performance and emission parameters using methanol/ND/BD blends, which can be of significant novelty.

The presented work explores PI mode in a diesel engine with a prime objective of reducing NO_x_ emissions and improving its performance. To mitigate the higher soot/smoke emissions associated with PI mode, 10% methanol was added by volume into base fuel, which is B30 (70% ND + 30% BD) and blend was named as M10. BD was included as a part of base fuel in order to ensure miscibility of methanol into ND. However, methanol addition into base fuel causes issues related to the burning of pilot fuel due to its higher ON and reduces the impact of PI mode. Due to this, injected pilot fuel may not burn completely before injection of main fuel and HTA available inside CC may not be sufficient to lower the DP of upcoming main fuel. Due to this, the objective behind implementing PI mode may not get fulfilled to the best possible extent. To address this problem, DTBP was used as CE and added into M10 in proportion of 0.5% by volume, and the blend was referred to as A1. The addition of CE into M10 compensates the reduction in CN of blend caused by the methanol addition and brings it close to CN of the base fuel. Because of this, issues related to burning of pre‐injected pilot fuel can be resolved successfully. PI mode was explored by varying pilot injection ratio (PIR) as well as pilot injection timing (PIT) using base fuel (B30), M10, and A1 blends along with comparison of all three blends.

## 2 Experimental Set Up and Research Procedure

### 2.1 Test Fuel

Waste cooking oil (WCO) was used as the feedstock for producing BD used in the present work, which was collected from the institute canteen (ground nut oil is used in the canteen). The suspended particles from the obtained oil were removed by sedimentation and vacuum filtering through linen cloth. Methanol and potassium hydroxide being used for transesterification reaction were of 99.9% purity which were supplied by Merck in Mumbai, India.

### 2.2 BD Production Process

**FIGURE 1:**
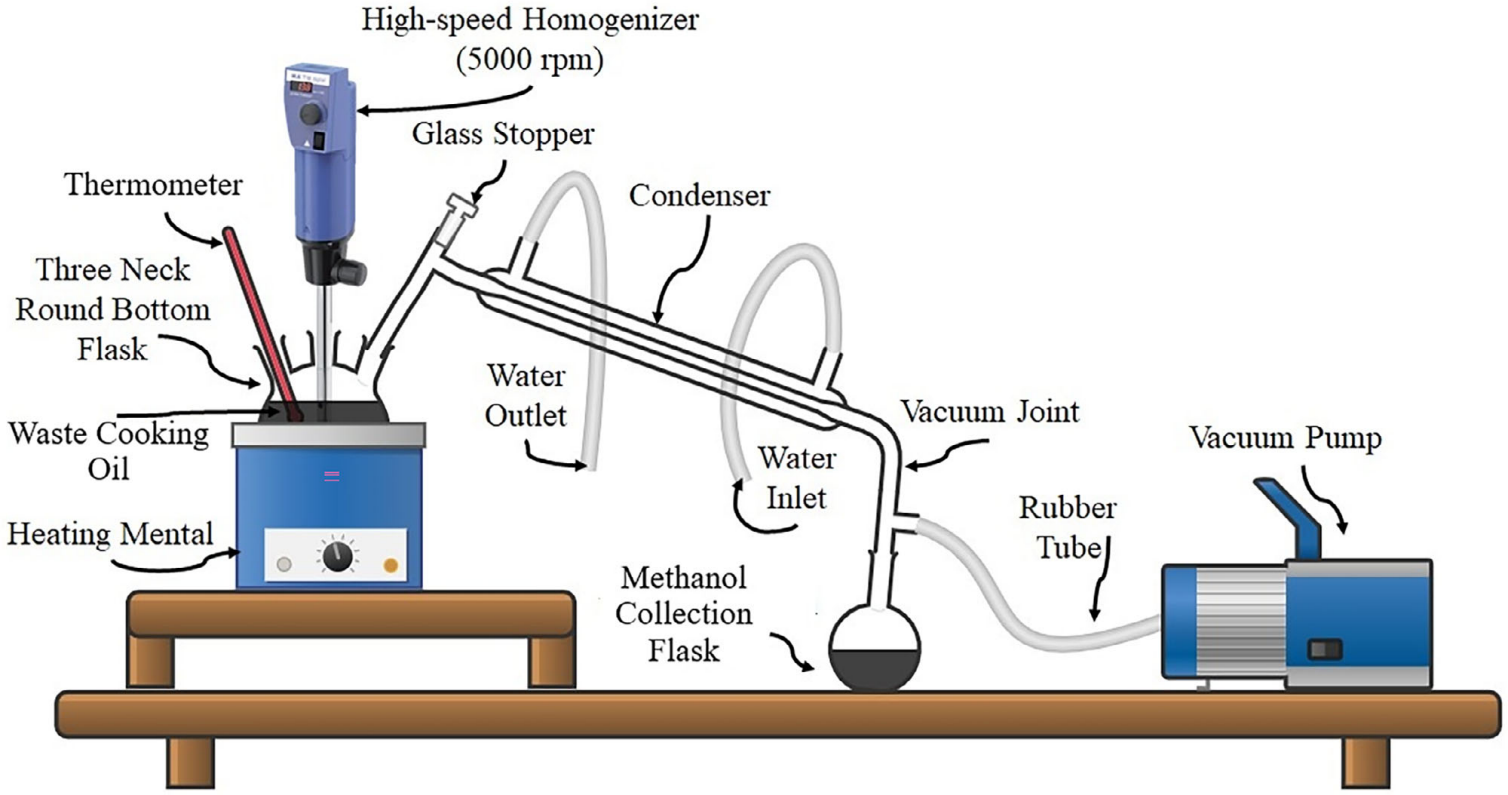
Line Diagram of Transesterification Reaction.

BD was produced from WCO using a transesterification reaction. A representative line diagram of set up used for the transesterification reaction is shown in Figure 1.

Transesterification reaction was carried out in a three necked 2‐L round bottom flask. A thermometer was mounted into one side of neck to monitor the reaction temperature, while a reflux condenser was fitted in the other side neck was to control evaporation of methanol. A heating mental integrated in the set up maintains reaction temperature constant at 70°C. Initially, mixture of filtered WCO and premeasured quantity of potassium hydroxide catalyst (1.2% by weight of the oil) dissolved in methanol was introduced inside reactor through one of the sides. The oil‐to‐methanol molar ratio of 1:6 was maintained for the reaction. The mixture was stirred using a high‐speed homogenizer at 1000 rpm for 60 min to ensure thorough mixing of the reactants. The product was cooled and taken to a separating funnel after completion of reaction where it was kept undisturbed for 24 h. Two separate layers of BD and glycerol were formed at the top and bottom of funnel respectively. Then after BD layer was separated, washed and dried at 95°C in order to meet its moisture content requirement in accordance with ASTM D6751 standard.

### 2.3 Blend Preparation

After getting BD, B30 (30% BD + 70% ND) was prepared using high speed homogenizer at 1000 rpm at 40°C to ensure homogeneous mixture. B30 is serving as a base fuel for this study. Then after, methanol was added by 10% in vol. in B30 and blend was named as M10.#x000A0;10% methanol by volume is significant enough to cause noteworthy elongation of the DP of base fuel (B30) without offering miscibility issues. M10 was prepared using a high‐speed homogenizer rotated at 3000 rpm for 45 min at 60°C temperature (using similar set up shown in Figure 1) to ensure miscibility of methanol into base fuel (B30). A condenser was used to prevent evaporation of methanol during blend preparation. CE used is DTBP which was blended with M10 in proportion of 0.5% by volume and blend was referred as A1. It is important to note that 0.5% DTBP by volume in M10 brings its CN close to base fuel which was decided based on start of combustion (SOC) angle. All the blends were kept under observation for 48 hr during which no phase separation was noticed. Then after they were used for actual engine testing. The properties of various fuels/additives used in presented work as well as details of blend are provided in Tables 1 and 2, respectively. Properties of different fuel blends are presented in Table 3.

**TABLE 1:** Fuel Properties.

Property	ND	BD	Methanol	DTBP
Density (kg/m^3^)	827	889	792	796
Kinematic Viscosity (mm^2^/s)	2.8	4.85	0.58	1.26
Oxygen (%)	—	10.86	50	21.90
Carbon (%)	86.15	76.92	37.50	65.69
Hydrogen (%)	13.85	12.22	12.50	12.41
Lower calorific value (MJ/kg)	41.25	38.86	20.10	33.80
Latent heat of vaporization (kJ/kg)	260	213	1162.50	264.20

**TABLE 2:** Nomenclature of the test fuel/fuel blends.

Blend No.	Name	Composition (by volume)
1	B30	70% Neat Diesel + 30% Biodiesel (Base fuel)
2	M10	90% B30 +10% Methanol
3	A1	89.5% B30 +10% Methanol+0.5% DTBP

**TABLE 3:** Properties of the blended fuels.

Property	B30	M10	A1
Density (kg/m^3^)	849	844	843
Kinematic Viscosity (mm^2^/s)	3.52	3.32	3.41
Lower calorific value (MJ/kg)	40.82	38.80	38.55
Latent heat of vaporization (kJ/kg)	246.30	338.50	324.60

### 2.4 Engine Test Rig

**FIGURE 2:**
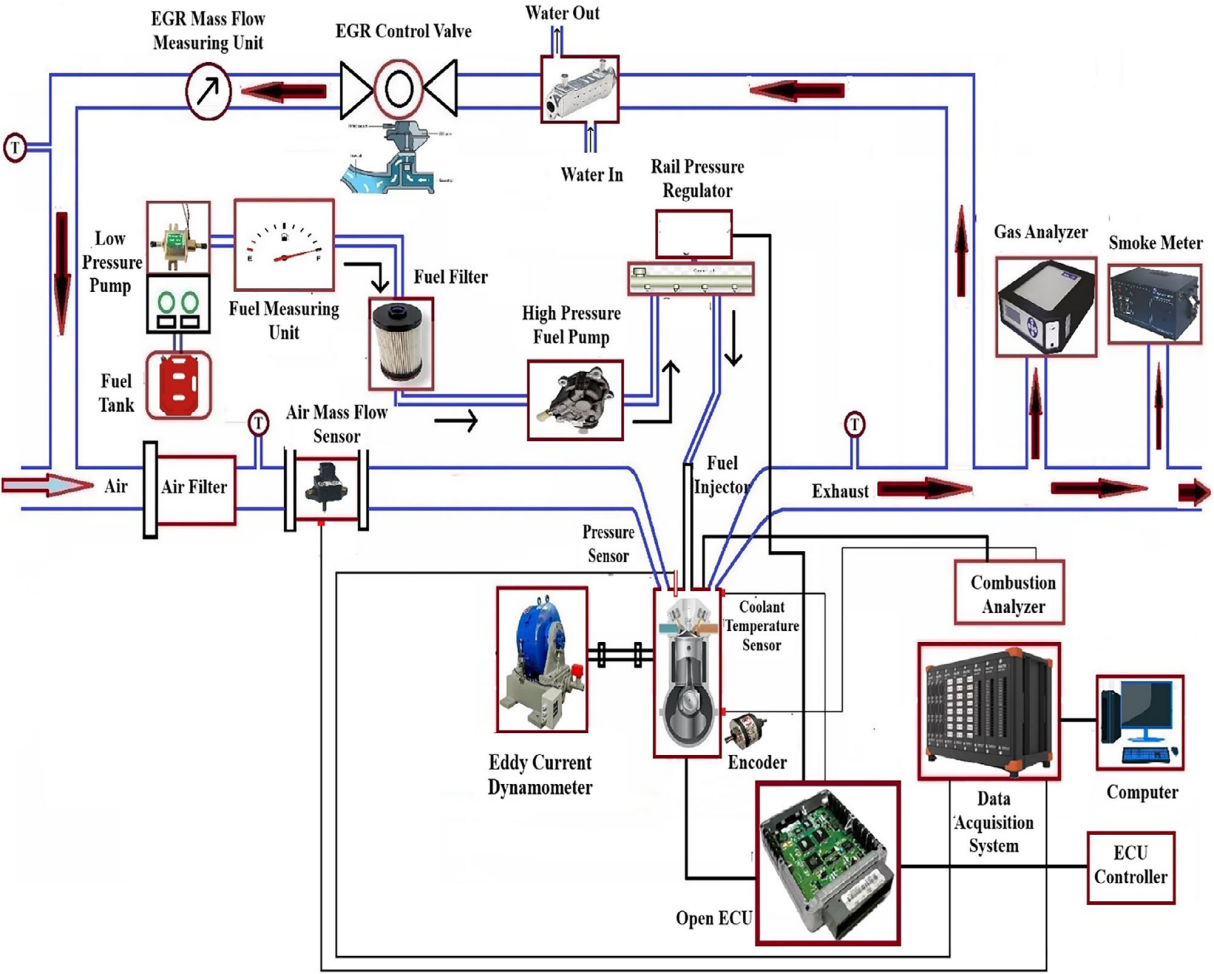
Schematic of the engine test rig.

The schematic of the engine test rig used for the present investigation is shown in Figure 2. The test engine is a Mahindra Jeeto mono‐cylinder diesel engine equipped with an eddy current dynamometer for loading it. Table 4 highlights the important specifications of the test engine along with fuel injection system.

**TABLE 4:** Specifications of the test engine and fuel injection system.

General details	Naturally aspirated, Direct injection, Water cooled
Swept volume (cc)	625
Compression ratio	18
Rated power (kW@rpm)	10@3600
Stroke length (mm)	92
Bore diameter (mm)	93
Piston bowl geometry	Re‐entrant
Rated torque (N‐m@rpm)	40@1600
Injection system	Common rail direct injection
Fuel injector	Solenoid type having 6 equally spaced nozzles
Fuel injection pressure range (bar)	200–1000
Maximum number of injections per cycle	3

A piezoelectric pressure transducer was employed to monitor the pressure inside the combustion chamber, which measures CGP at each 1° crank angle (CA) interval. An encoder was used to measure the position of crank shaft. To minimize cyclic variations, CGP was recorded for 100 consecutive cycles, and their average value was considered as CGP at each CA. Various combustion parameters used in the study were acquired using measured CGP data. Moreover, sensors for measuring fuel flow, air flow as well as temperature are also placed in the test rig. A data acquisition system captures signals from various sensors of the test rig, which are directly communicated to a computer system. An open ECU was utilized to control the fuel injection related parameters like injection pressure, injection timing, number of injections per cycle as well as fuel mass distribution in each injection. AVL 444N gas analyzer was used for measuring HC, CO, and NO_x_ emissions while smoke emissions in the form of smoke opacity were recorded using AVL 437 smoke meter. As the facility to measure particulate matter (PM)/soot emissions is not available, smoke opacity was measured, which represents density of soot particles.

### 2.5 Uncertainty Analysis

Uncertainty analysis was carried out to minimize errors in measurement of various parameters, which can be due to factors like environment conditions, errors in measurement process, sensor accuracy, etc. All the sensors were calibrated before experiments to minimize calibration errors. Also, measurement errors were reduced by conducting experiments three times and considering average values. Table 5 highlights the details of various measuring devices of the test rig and their uncertainty.

**TABLE 5:** Details of instruments along with their uncertainty.

Parameter	Instrument; Make	Uncertainty
Air flow rate	Mass flow sensor; Bosch	± 1%
Engine load	Load Cell; Sensotronics	± 0.075 kg
Cylinder pressure	Pressure Transducer; PCB	± 1%
Fuel flow rate	Mass flow sensor; Dawyer	± 0.5%
NO_x_	Flue Gas Analyzer; AVL 444N	± 1%
CO		± 3%
HC		± 3%
Smoke	Smoke Meter; AVL 437	± 1%

Uncertainty of brake power (BP), BSFC, and BTE was found as per Equation (1).

If y = f (x_1_, x_2_, x_3_…x_n_), then uncertainty (*U_y_*) is given as

(1) $$U_{y}=\pm {\left[\sum_{i=1}^{n}{\left(\frac{\partial y}{\partial x_{i}}\right)}^{2}{\left(U_{x_{i}}\right)}^{2}\right]}^{0.5}$$

where *U_xi_* is the uncertainty of the parameter *x_i_*.

The calculated value of uncertainty for BP, BSFC, and BTE is ± 0.072%, ± 0.86%, and ± 0.98%, respectively.

### 2.6 Experimental Operating Conditions

The engine was operated at a constant speed of 1600 rpm (rated torque speed) and constant load of 90% of full load during all the tests. Also, the fuel injection pressure was kept constant at 500 bar throughout the tests. Single injection at −15° after top dead center (ATDC) injection timing with base fuel (B30) served as a reference condition. This injection timing (−15° ATDC) obtained the combination of highest indicated power (IP) and lowest BSFC during injection timing sweep from −30° ATDC to −5° ATDC in the interval of 5°CA.

The considered variables in the case of PI modes are PIR and PIT while main injection timing was held constant at −15° ATDC. PIR was varied from 10% to 30% of total fuel mass per cycle in the interval of 10% while holding PIT constant at −30° ATDC. The minimum PIR considered here (10%) is sufficient to create HTA inside CC before main injection. PIT varied from −24° ATDC to −36° ATDC in steps of 6°CA interval while maintaining constant PIR of 10%. More details regarding experimental operating conditions are provided in Table 6.

**TABLE 6:** Experimental operating conditions.

Run no.	Type of injection	Main injection timing (° ATDC)	PQ (%)	PIT (° ATDC)
1	Single	−15	0	—
2	Pilot + Main	−15	10	−30
3	Pilot + Main	−15	20	−30
4	Pilot + Main	−15	30	−30
5	Pilot + Main	−15	10	−24
6	Pilot + Main	−15	10	−36

## 3 Results and Discussion

This section represents a brief analysis of major combustion, performance emission characteristics of the test engine using three blends namely B30, M10 as well as A1, respectively. Comparison of the characteristics obtained using these three blends was made under various PI modes. Also, PI mode was compared with SI mode.

### 3.1 Combustion Characteristics

**FIGURE 3:**
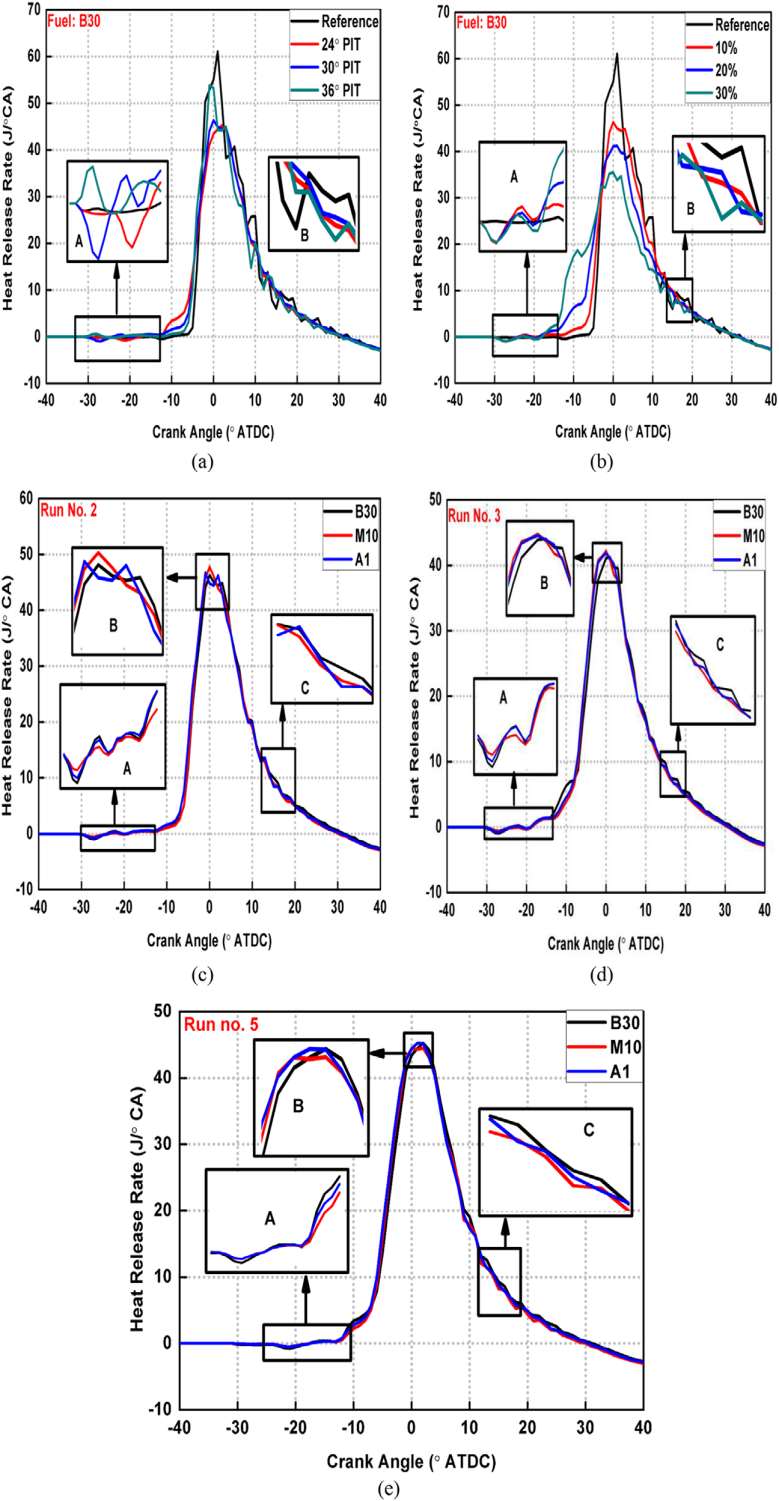
HRR traces for (a) varying PIT (b) varying PIR (c–e) various test fuels.

Figure 3 shows the HRR traces for various blends under different PI modes.

Figure 3a,b compare various PI modes with single injection, which is also a reference condition using base fuel (B30) under varying PIT and PIR conditions, respectively. PI mode reduced peak HRR significantly as seen from Figure 3a,b. This is owing to HTA formulated inside CC due to burning of pilot fuel as highlighted in expanded view (part A) causing shorter main fuel DP and peak HRR reduced subsequently. Earlier PIT as well as smaller PIR produced greater premixed and lower diffusion combustion peaks respectively for pilot fuel (part A) as visible from Figure 3a,b. This is thought to be due to longer DP of pilot fuel associated with earlier PIT. In case of greater PIR, DP gets shortened because of greater rich zones due to injection of larger pilot fuel quantity causing earlier SOC for pilot fuel. Also, the pre‐mixed peak for main fuel is lower in case of retarded PIT and greater PIR as illustrated in Figure 3a,b, respectively. The HTA produced by pilot fuel burning shifts closer to main fuel in case of retarded PIT (as main injection timing is held constant) causing it to burn earlier by shortening its DP. In case of greater PIR (Figure 3b), HTA is bigger due to burning of larger PIR (part A in Figure 3b). Also, this bigger HTA shifts closer to main fuel due to greater injection duration associated with greater PIR (due to constant main injection timing). Also, main fuel mass was reduced proportionally with increasing PIR. All these three factors shortened DP of main fuel more effectively for the case of greater PIR and subsequent lower pre‐mixed peak obtained for main fuel. The shorter DP and subsequent reduced mixing time of main fuel for the cases of retarded PIT as well as larger PIR produced earlier, and greater main fuel diffusion peaks (part B) as seen from Figure 3a,b, respectively. Also, the diffusion peak lowered and shifted later for SI case compared to all the PI modes as shown in (part B) Figure 3a,b. This because of the unavailability of HTA in case of SI mode, which elongated its DP resulting in more homogenous air‐fuel mixture by offering sufficient mixing time.

Figure 3c–e represents HRR traces with different fuel blends under similar PI modes. Addition of methanol into base fuel (M10 blend) reduced HTA compared to B30 as shown in (part A of) Figure 3c–e. This is because of the increased ON of M10 blend due to methanol addition increasing its self‐ignition temperature (SIT) and hindering the burning of pilot fuel. This resulted in longer main fuel DP for M10 blend compared to B30 and subsequent greater pre‐mixed peak (part B) as well as shorter diffusion peaks (part C) for main fuel were obtained as seen from Figure 3c–e. When 0.5% DTBP was added into the M10 blend (causing A1 blend), HTA became almost like base fuel as shown in (part A of) Figure 3c–e. This is because the addition of CE (DTBP for the present work) into the M10 blend brought its CN nearly equal to base fuel. It is also evident that 0.5% DTBP by volume in M10 blend is the quantity that increases CN of M10 blend and brings it nearly equal to CN of B30 fuel as discussed in section 2.3.

**FIGURE 4:**
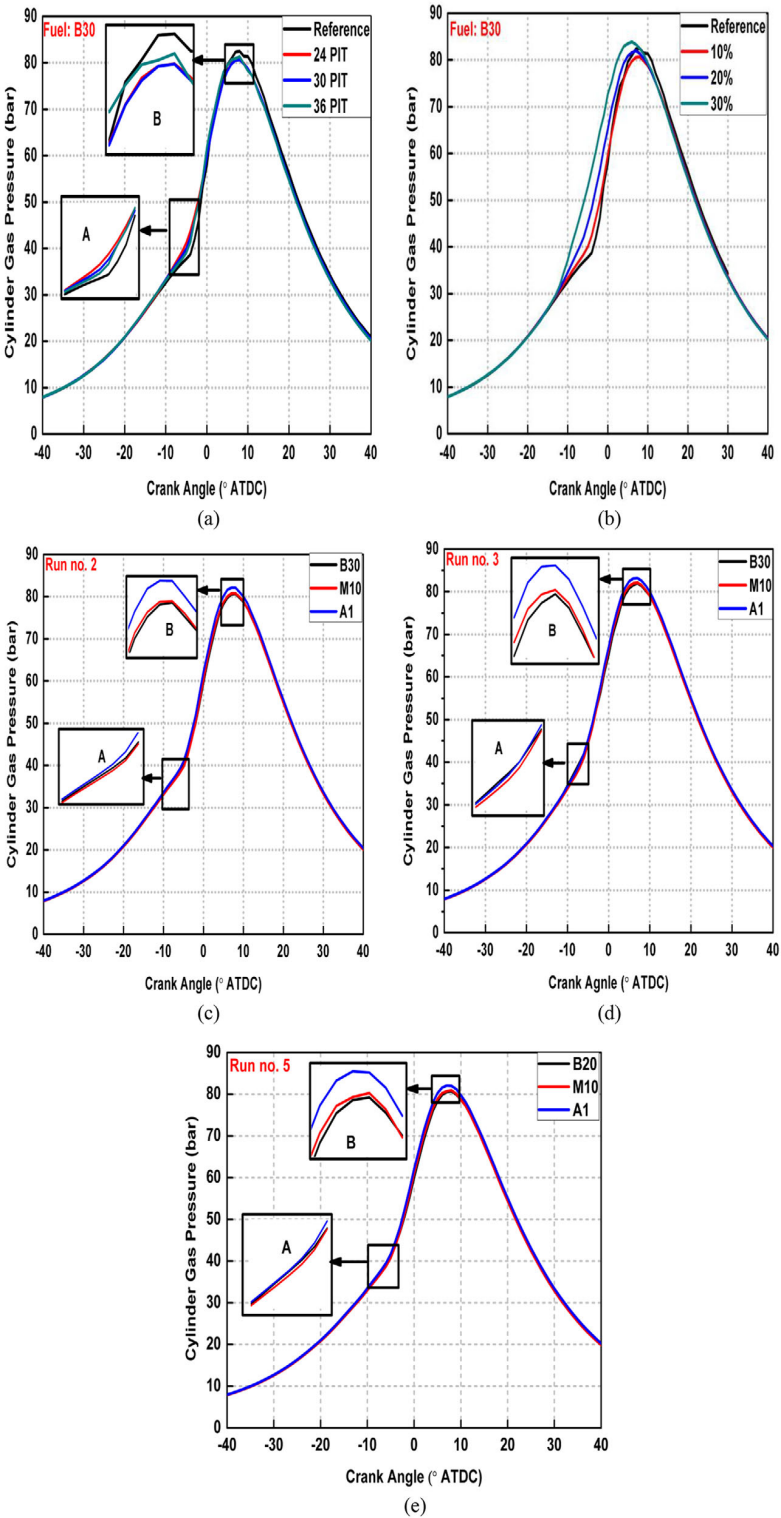
CGP traces for (a) varying PIT (b) varying PIR (c–e) various test fuels.

Figure 4 represents CGP curves for various blends under different PI modes. Figure 4a,b represent the effect of varying PIT and varying PIR on CGP traces along with Run no. 1 (SI mode) for base fuel. CGP starts increasing much earlier in case of PI modes compared to the SI mode as shown in (part A) Figure 4a,b. This is owing to HTA produced by burning pilot fuel before main injection. Also, peak CGP shifts earlier and gets lower for all the PI modes compared to the SI mode except for the cases of 20% and 30% PIR. This is because of the availability of HTA in case of PI modes causing less severe pre‐mixed phase of main fuel (Figure 3a,b) and reduced constant‐volume burning proportion of main fuel which led to lower CGP compared to SI mode. The HTA starts burning main fuel earlier and shifts overall combustion process toward TDC. Because of this peak CGP shifted earlier and approached the ideal diesel cycle more closely indicating improved combustion process. Also, peak CGP increased for the case of −36° ATDC PIT compared to other two PITs for which it remained almost similar as seen from Figure 4a. This is because in the case of −36° ATDC PIT, pilot fuel was injected in less favorable thermodynamic conditions for auto‐ignition compared to other two PITs and produced smaller HTA (Figure 3a), which was also away from main fuel. Due to this, DP for main fuel was reduced less effectively and greater pre‐mixed phase of main fuel was obtained (Figure 3a) compared to other two PITs and led to greater CGP. Also, peak CGP increased with greater PIR as shown in Figure 4b. This is because of the greater HTA formed (Figure 3b) and placement of this greater HTA nearer to main fuel with increasing PIR. Both these factors started burning the main fuel earlier with an increase in PIR and started increasing CGP much earlier.

Also, M10 blend started rising CGP somewhat later (part A) and produced slightly higher CGP (part B) compared to B30 as shown in Figure 4c–e. These effects are more clearly visible for Run nos. 3 and 5 compared to Run no. 2. The late start of CGP rise is attributed to higher ON of methanol, which caused longer DP and subsequent greater fuel mass combusted in premixed phase (Figure 3c–e) strengthening constant volume proportion in cycle and led to higher peak CGP. Also, significant oxygen content of methanol played a vital role for getting higher peak CGP. Similar results were reported by references [10, 44]. Additionally, when DTBP was added (A1 blend), CGP started rising much earlier and peak CGP was also much higher compared to both M10 blend. For example, peak CGP rose by 1.63% in case of A1 blend compared to M10 for Run no. 2. This is because of the higher CN of A1 blend compared to M10 blend, which started burning fuel earlier and maintained overall high CGP inside CC.

**FIGURE 5:**
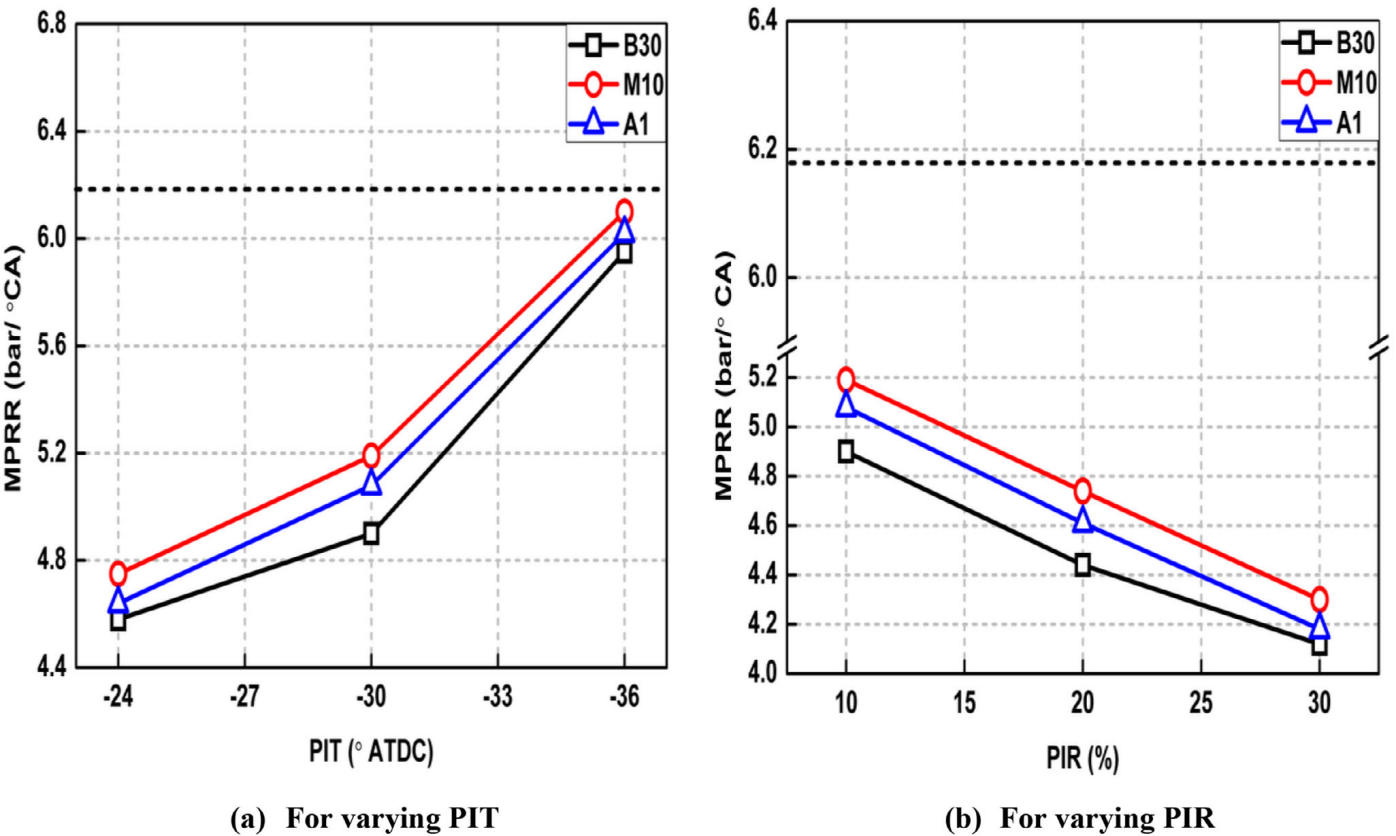
Effect of various fuel blends on MPRR under PI modes.

Effect of all the tested fuel on the maximum pressure rise rate (MPRR) under PI modes is illustrated in Figure 5 along with reference condition (represented as horizontal dashed lines).

MPRR significantly depends upon the rate of combustion/burning of fuel. Faster fuel burning rates, which are realized during pre‐mixed combustion phase (where fuel burns instantaneously) lead to greater MPRR and vice versa. MPRR is notably lower in the case of SI mode compared to all PI modes. For example, MPRR reduces by 1.29 bar/°CA for Run no. 2 compared to Run no. 1 (reference condition). This is because HTA produced by burning of pilot fuel lowers the DP of main fuel, which reduces the severity of the pre‐mixed phase for main fuel (Figure 3a,b). Retarded PIT and greater PIR resulted in lower MPRR as shown in Figure 5a,b, respectively as both these led to lower pre‐mixed combustion phase as visible in Figure 3a,b, respectively. Also, M10 produced higher MPRR than B30 which is attributed to higher ON of M10 compared to B30, which prolonged the DP and strengthened pre‐mixed phase. When DTBP was added, again CN of M10 lowered and MPRR was reduced in case of A1 blend compared to M10 as shown in Figure 5.

**FIGURE 6:**
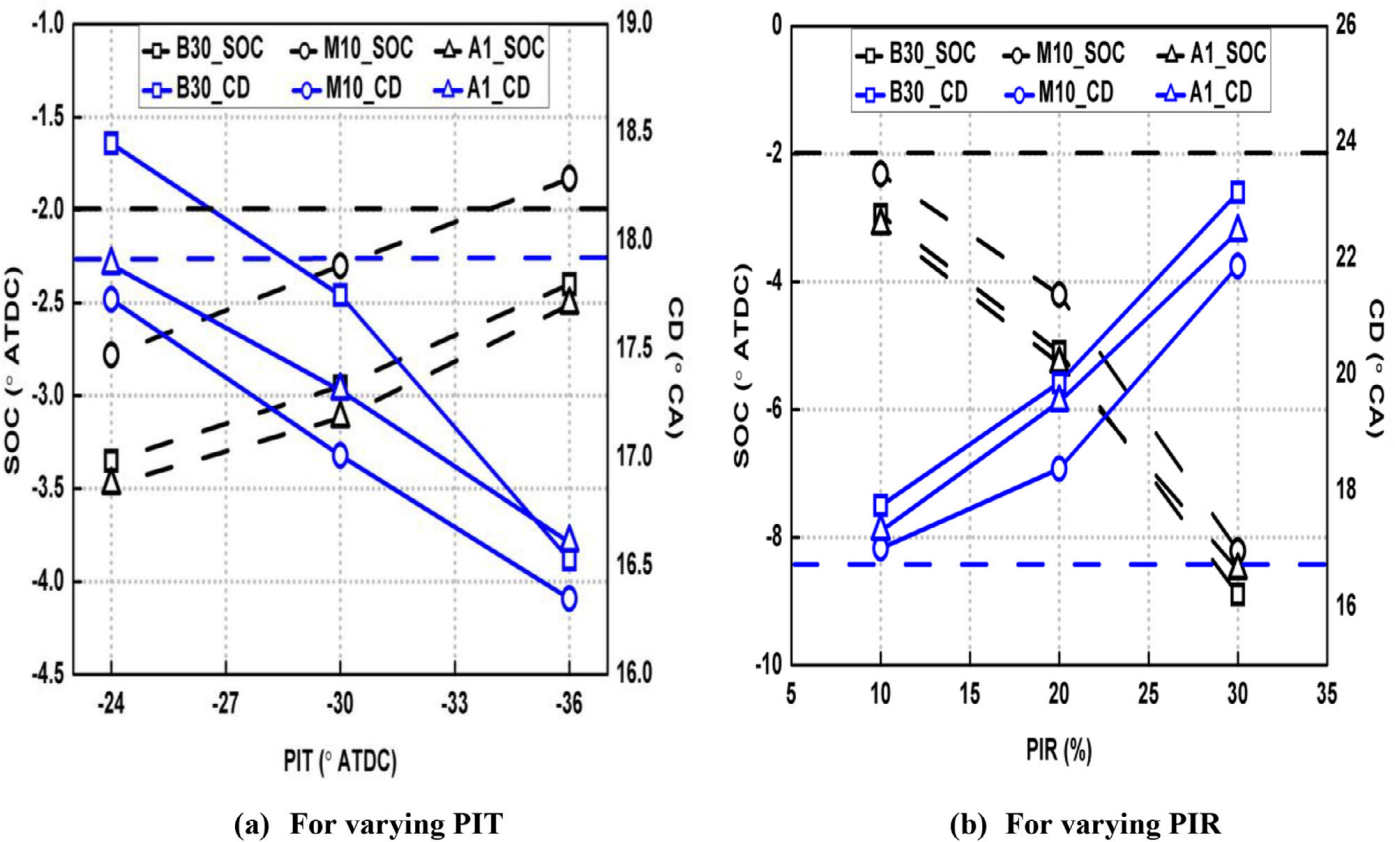
Effect of various fuel blends on SOC and CD under PI modes.

Figure 6 represents the effect of various fuel blends on SOC and combustion duration (CD). The CAs correspond to 10% and 90% of overall heat release were taken as SOC and end of combustion (EOC) respectively. CD was obtained by subtracting CA10 from CA90.

Implementing PI mode offered a significant advancement of SOC. For example, combustion starts earlier by 0.96°CA for Run no. 2 compared to the SI mode for base fuel. This is due to HTA being produced by combustion of pilot fuel as discussed earlier. Retarded PIT as well as higher PIR resulted in earlier SOC as seen from Figure 6a,b, respectively. This is because of shifting of produced HTA closer to main injection for the cases of closer pilot injection as well as greater PIR, which impacted it in a better manner. Also, the M10 blend retarded SOC compared to B30. For example, M10 blend retarded SOC by 0.65°CA compared to B30 for Run no. 2. This is because of higher ON of methanol present in M10 blend, which increased its DP compared to B30 and delayed its combustion. However, the A1 blend brought SOC nearly equal to base fuel due to increased CN of blend due to the presence of DTBP. It is important to note from Figure 6 that SOC is almost similar for the base fuel and A1 blends, which proves that 0.5% DTBP is the required quantity that increases CN of M10 blend such that it becomes nearly equal to CN of base fuel.

Also, CD reduces when PI mode is activated compared to SI case except for the case of closest pilot injection (−24° ATDC PIT) as seen from Figure 6. For example, CD was reduced by 1.08°CA for Run no. 2 compared to the SI mode for base fuel. This is caused by the shorter main fuel DP under PI mode, which subsequently caused less fuel to burn during pre‐mixed mode (where fuel burns instantaneously) and more fuel combusted during diffusion mode (where fuel burns gradually) compared to the SI case (Figure 3a,b) and overall CD elongated. Also, DP gets shortened with retardation of PIT as well as increasing PIR and led to longer CD as visible from Figure 6a,b, respectively. In case of M10 blend, CD reduced by 0.74°CA for Run no. 2 compared to B30 fuel. This is because of higher ON of M10 blend (due to presence of methanol) compared to B30 which offered longer DP, and more fuel mass participated in pre‐mixed phase and CD gets lowered. However, addition of DTBP into M10 blend (forming A1 blend) again reduced DP due to increased CN and CD was prolonged compared to M10 blend.

### 3.2 Performance and Emission Characteristics

**FIGURE 7:**
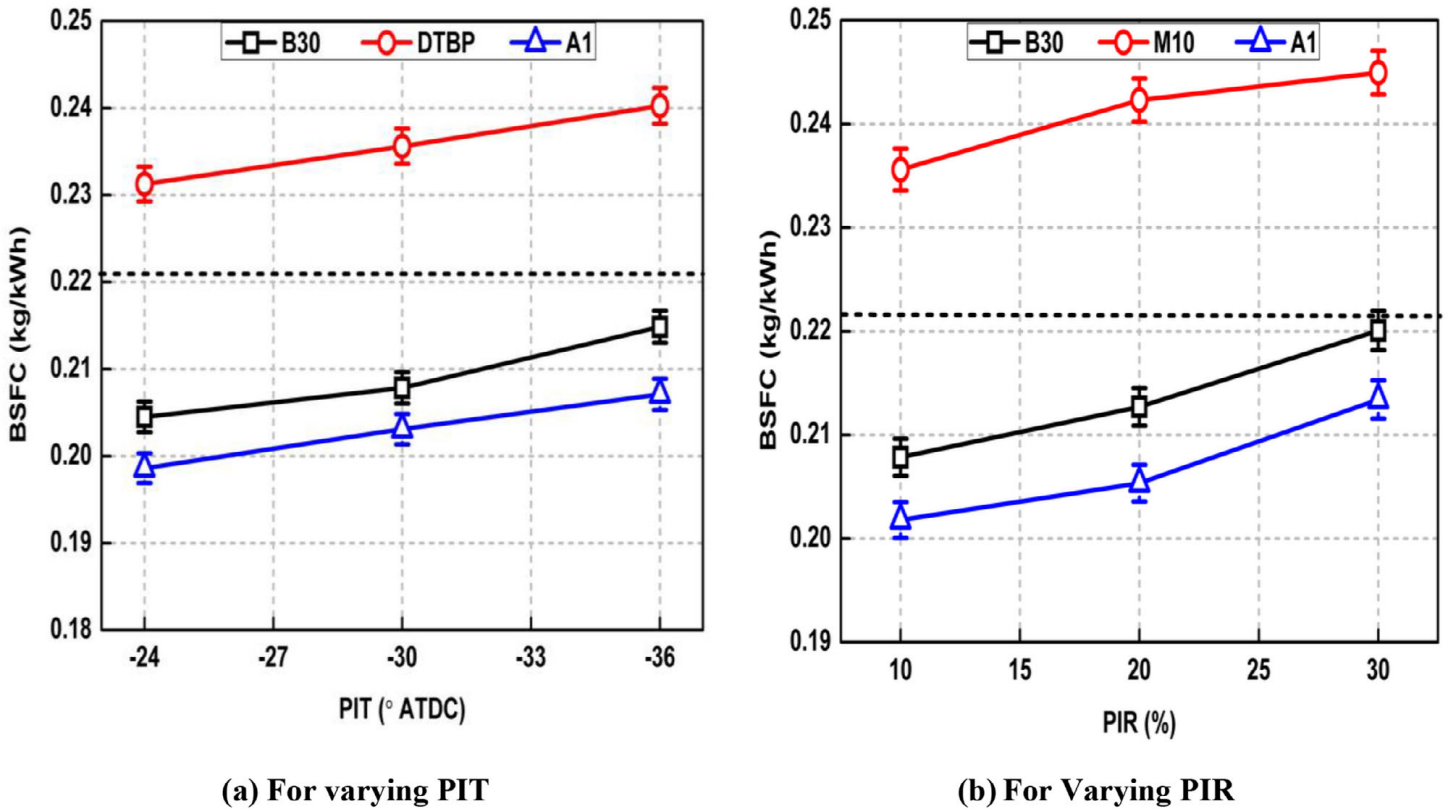
Effect of various PI modes on BSFC for the tested fuels.

**FIGURE 8:**
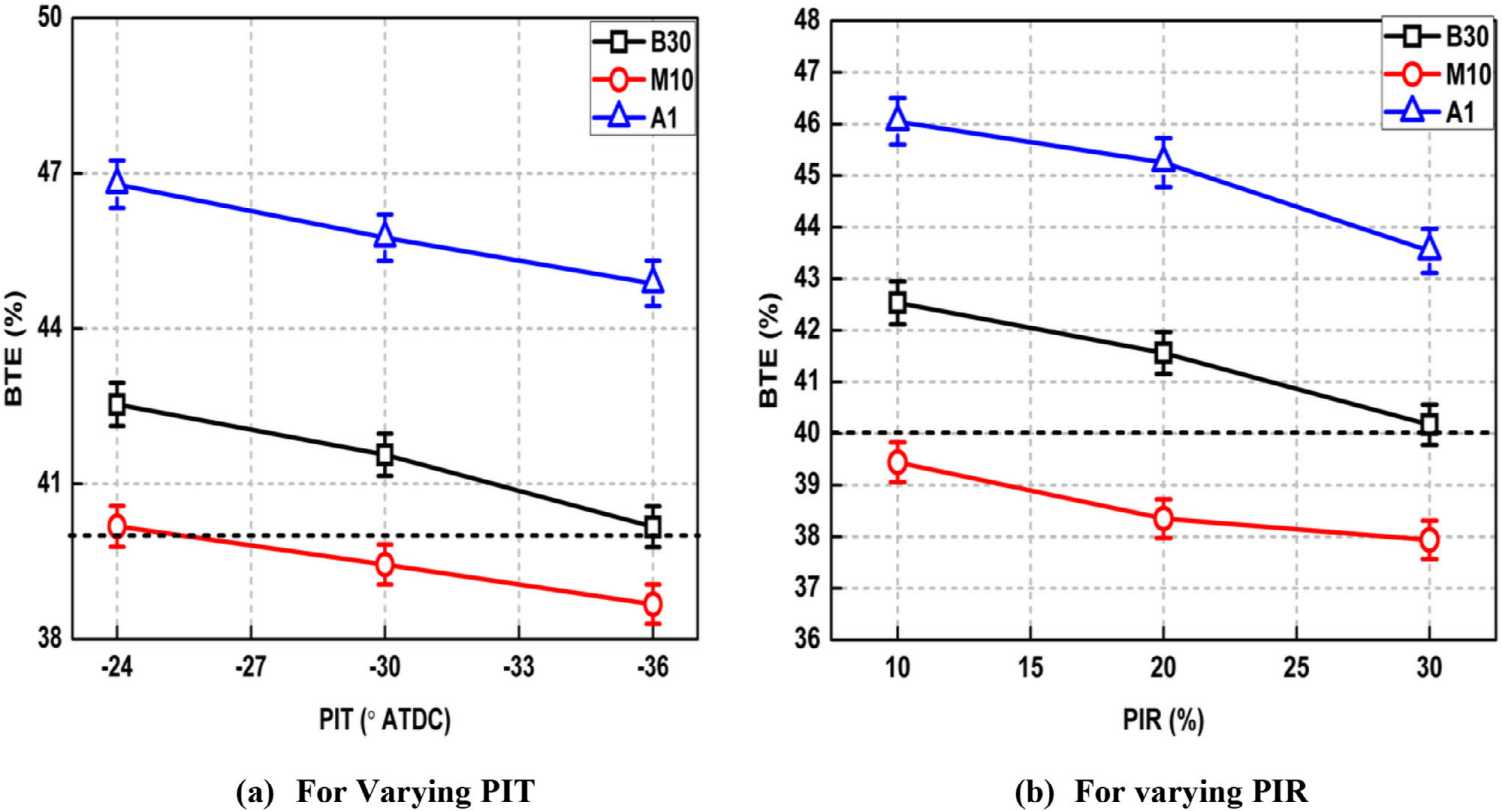
Effect of various test fuels on BTE under different PI modes.

Figures 7 and 8 show the effect of various PI modes on BSFC and BTE respectively for all the test fuels along with SI mode. BSFC represents the mass flow of fuel required to produce unit BP while the ability of an engine to convert fuel's total (chemical) energy into mechanical work is given by BTE. Both these performance parameters, largely dependent on fuel injected mass per cycle to attain given BP (which is constant for all the tests) as well as calorific value of fuel.

BSFC and BTE improved in case of PI modes compared to SI mode for base fuel. For example, BSFC and BTE improved by 5.08% and 5.98% respectively in case of Run no.2 compared to Run no. 1 for the case of base fuel. This is attributed to the improved combustion process due to shifting of peak CGP toward TDC as discussed earlier. Also, retarded PIT and lower PIR led to lower BSFC and higher BTE as seen from Figures 7 and 8, respectively. BSFC dropped by 4.83% and 5.58%, while BTE increased by 4.83% and 5.55% when PIT is retarded from −36° ATDC to −24° ATDC PIT and PIR reduced from 30% to 10%, respectively for the base fuel. This is owing to shifting of HTA nearer to main injection with retarded PIT, which linked pilot and main fuel combustion events more smoothly resulting in improved BSFC and BTE. For the case of PIR variation, increasing PIR started increasing CGP earlier (Figure 4b) and the negative work on piston was increased offering BSFC penalty with increasing PIR.

The addition of methanol in B30 blend (forming M10 blend) led to higher BSFC and lower BTE, respectively. This is in‐line with references [10, 24]. For example, BSFC increased by 9.85% and BTE reduced by 7.27% in case of M10 blend compared to B30 for Run no. 2. This is thought to be due to lower CV of methanol as well as its higher latent heat of vaporization compared to base fuel, and more fuel was required to be injected to attain similar BP which deteriorated BSFC as well as BTE. The A1 blend offered a significantly lower BSFC and higher BTE than M10 blend as seen from Figures 7 and 8 respectively. This is also evident from Figure 4c–e where higher peak CGP is observed for A1 blend compared to M10 blend indicating greater work output from the thermodynamic cycle. The reason for the improved BSFC as well as BTE in case of A1 blend compared to M10 is shifting of combustion process toward TDC due to earlier SOC for A1 blend, which improved overall combustion phenomena.

**FIGURE 9:**
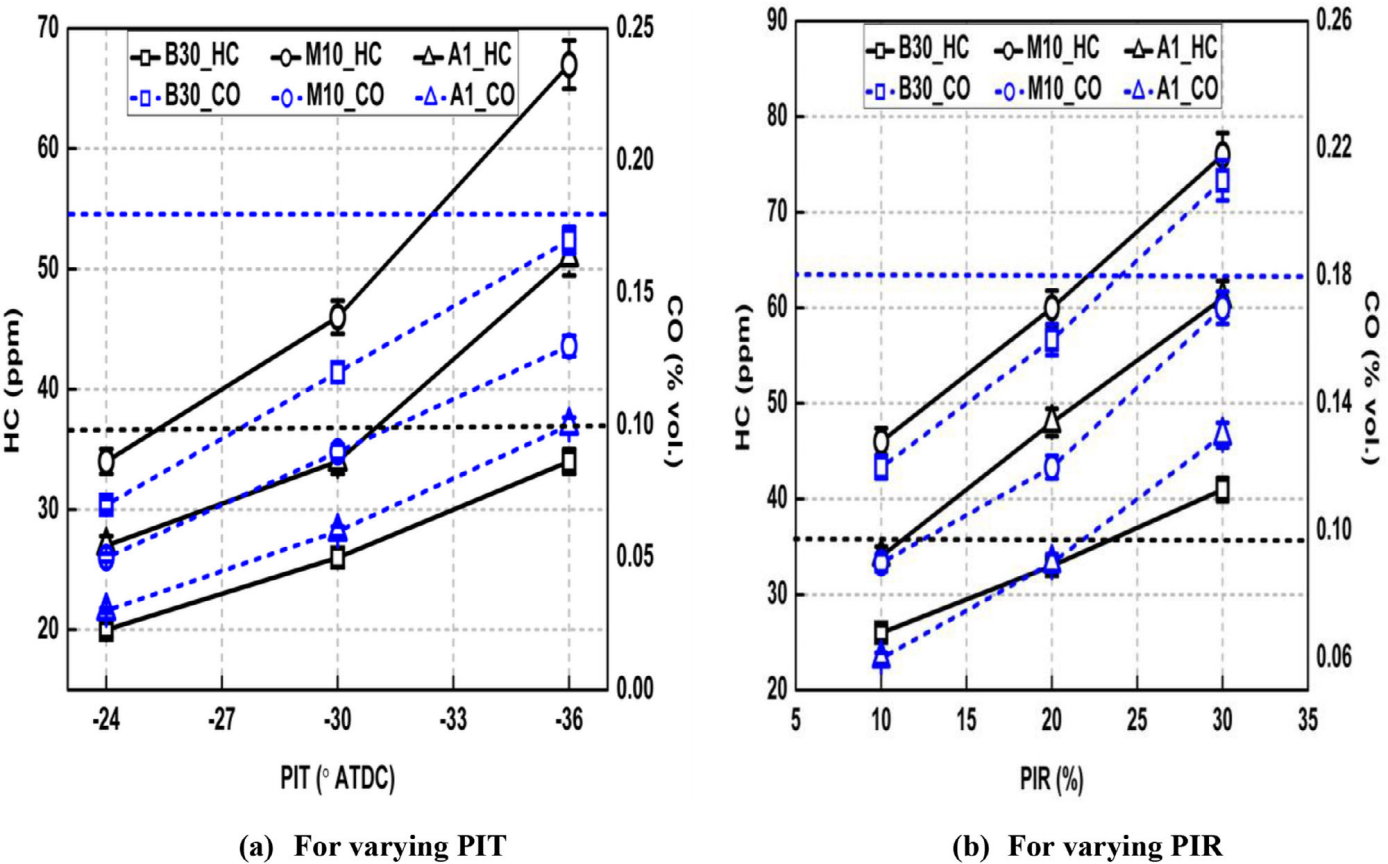
Variation of HC and CO emissions under different PI modes.

The variation of HC and CO emissions for each fuel is discerned in Figure 9.

HC and CO emissions form due to incomplete combustion. Injection of pilot fuel reduced both these emissions compared to SI case as seen from Figure 9. For example, Run no. 2 offered reduction in HC and CO emissions by 29.72% and 33.33% compared to Run no. 1 for the case of base fuel. This is owing to improved combustion process in case of PI modes which burnt fuel more completely. Also, advanced PIT and larger PIR resulted in higher HC as well as CO emissions. For example, HC emissions climbed by 41.18% and 36.58%, while CO emissions increased by 58.52% and 42.82% when PIT advances from −24° ATDC to −36° ATDC and PIR increases from 10% to 30%, respectively for the base fuel. Advanced PIT as well as greater PIR both cause increased wall wetting due to lesser charge density inside cylinder and greater momentum of spray, respectively. Also, DP of pilot fuel increases with advancement of PIT as well as increasing PIR which caused more fuel to approach crevice regions where its burning became difficult. Both these factors, that is, increased wall wetting as well as longer DP associated with early PIT and larger PIR led to higher HC and CO emissions.

M10 blend increased HC emissions while lowering CO emissions compared to B30. HC emissions increased by 43.47% and CO emissions were reduced by 25.12% in case of M10 compared to B30 for Run no. 2. Despite higher oxygen content of M10 blend, lower air‐fuel ratio due to injection of more fuel mass to attain similar BP can be the main reason for attaining higher HC emissions compared to B30. Also, the DP of pilot fuel gets elongated due to higher ON of M10 blend compared to B30 allowing greater pilot mass to approach the crevice regions and HC emissions increased. However, HC emissions lowered for the A1 blend compared to M10 as shown in Figure 9. This is again due to shortened DP of the A1 blend due to its higher CN compared to M10.

The reduction in CO emission in case of M10 blend compared to B30 is primarily due to lower carbon content and higher oxygen content of methanol which reduced local rich zones (where majority of CO forms) and improved post flame oxidation of formed CO [45]. Also, the addition of DTBP into M10 blend reduced CO emission which is mainly due to (i) improved combustion process due to presence of CI (indicated higher peak CGP from Figure 4c–e) and (ii) presence of oxygen in DTBP.

**FIGURE 10:**
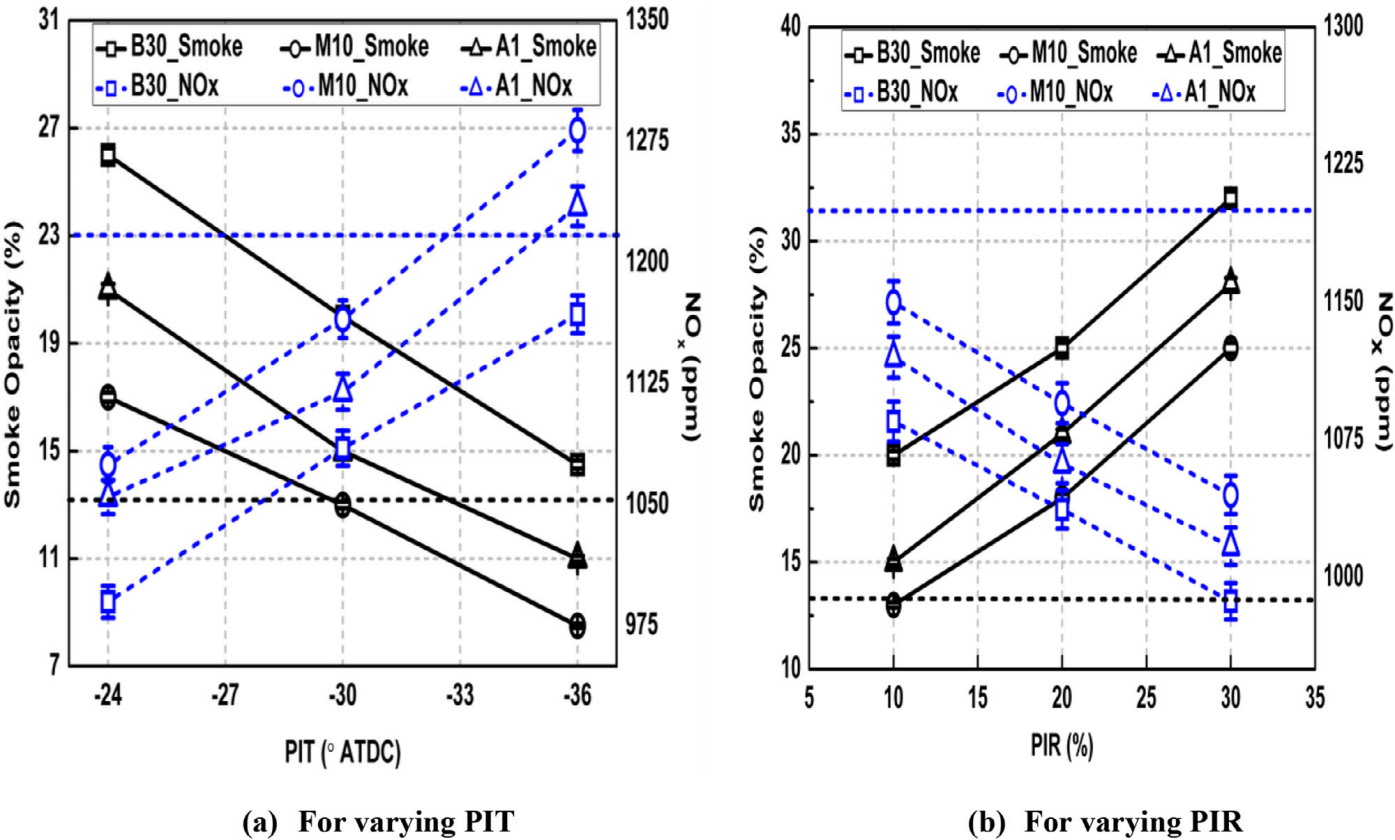
Variation of NOx and smoke emissions under different PI modes.

The variation of NO_x_ and smoke emissions for various fuels under all the tested conditions is shown in Figure 10.

PI mode offers noteworthy reduction in NO_x_ emissions compared to SI mode while smoke emissions increased. NO_x_ emissions lowered by 9.36% and smoke emissions rose by 35.2% in the case of Run no. 2 compared to SI mode for base fuel. This due to shortened DP of main fuel under PI mode compared to SI mode because of HTA formed by burning of pilot fuel which lowered the severity of pre‐mixed phase and reduced peak HRR (Figure 3a,b) resulting in lower NO_x_ formation compared to SI mode. The shorter main fuel DP reduced mixing time for fuel and air to mix with each other and reduced homogeneity of mixture causing greater rich regions leading to greater soot formation. This is also evident by greater and earlier diffusion peaks under PI modes compared to SI mode from Figure 3a,b. Also, CD is longer in case of PI modes compared to SI mode (Figure 6) allowing lesser time for oxidation of formed soot particles at higher temperature causing higher net soot exhaust from the tail pipe. Retarded PIT and larger PIR resulted in lower NO_x_ and higher soot emissions respectively as shown in Figure 10a,b, respectively. For example, NO_x_ emissions dropped by 15.23% and 9.08%, while smoke emissions increased by 44.25% and 37.52% when PIT retarded from −36° ATDC to −24° ATDC and PIR increases from 10% to 30%, respectively for the base fuel. This is because both retarded PIT and larger PIR shortens the main fuel DP more effectively as discussed earlier.

M10 blend offers lower smoke emissions compared to B30 while increasing NO_x_ emissions. For Run no. 2, NO_x_ increased by 5.35% while smoke reduced by 34.89% in case of the M10 blend compared to base fuel. Similar results were reported by references [10, 46]. Therefore, it can be said that the rise in NO_x_ emissions due to the addition of methanol into B30 is much smaller compared to the drop obtained in smoke emissions. Also, it can be said that the methanol addition into base fuel successfully resolves the problem of higher smoke/soot emissions associated with PI mode. This is because of higher ON of M10 blend resulting in longer DP of main fuel as shown in Figure 6, which allowed longer mixing time for air‐fuel mixture preparation and formed mixture with higher homogeneity leading to higher NO_x_ and lower soot formation, respectively. Also, inherent oxygen content of methanol can enhance NO_x_ formation and support soot oxidation. Adding DTBP into M10 (causing A1 blend) again increased smoke emissions while lowering NO_x_ emissions. This is again due to higher CN of A1 blend compared to M10 causing reduction in DP and subsequent lesser air‐fuel mixing time.

## 4 Result Analysis

Table 7 represents a comparison of important parameters between reference condition (single injection (Run no. 1) with base fuel) and Run No. 2 using A1 fuel. Run no. 2 is selected for comparison purpose as this is the PI mode with lowest PIR (10%) which offers a noteworthy reduction in NO_x_ emissions (the main motive behind opting PI mode) compared to SI mode while offering minimal rise in smoke emissions among all PI modes.

**TABLE 7:** Comparison of important parameters.

Run no.	Blend	BTE (%)	BSFC (kg/kWh)	HC (ppm)	CO (%)	NO_x_ (ppm)	Smoke Opacity (%)
1	B30	39.98 ± 0.3918	0.2212 ± 0.0019	37 ± 1.11	0.18 ± 0.0054	1197 ± 11.97	13.1 ± 0.131
2	A1	46.04 ± 0.4512	0.2018 ± 0.0017	34 ± 1.02	0.06 ± 0.0018	1120 ± 11.20	13.45 ± 0.1345
**% change in 2 compared to 1**		13.16 **(↑)**	8.77 **(↓)**	8.11 **(↓)**	66.66 **(↓)**	6.43 **(↓)**	2.60 (↑)

It can be seen that combination of PI mode and A1 blend improves all the major emissions except smoke along with offering advantages of much better BSFC and BTE compared to SI mode with base fuel. From Table 7, it can be clearly pointed out that the rise in smoke emissions is very minimal compared to the drop NO_x_ as well as HC and CO emissions. Therefore, it can be said that addition of methanol and DTBP which is just enough to compensate the rise in ON of base fuel caused by methanol addition can successfully resolve the problem of higher smoke emissions coupled with PI mode while maintaining all the benefits of PI mode compared to the SI mode like better fuel economy, higher BTE as well as lower HC, CO and NO_x_ emissions.

## 5 Conclusions

The presented work is an effort in the direction of improving smoke emissions under PI mode in a diesel engine while maintaining the benefits of PI mode compared to SI mode. This was achieved by adding methanol and DTBP as additives to the base fuel, which is B30. Some important conclusions drawn from the conducted work are as follows:

- The HTA produced by burnt pilot fuel triggered the main combustion earlier and advanced the peak CGP causing improved engine combustion and performance. Also, PI mode is very effective in terms of reducing NO_x_, HC as well as CO emissions compared to SI mode. In case of base fuel, Run no. 2 improved BSFC and BTE by 5.08% and 5.98% respectively, compared to SI mode while reducing HC, CO and NO_x_ emissions by 29.73%, 33.33%, and 9.36% respectively. However, smoke emissions were elevated by 35.2%.
- The retarded PIT as well as smaller PIR enhanced fuel economy as well as thermal efficiency of the engine.
- Due to increased ON, M10 blend produced less severe HTA compared to B30 forming air‐fuel mixture with higher homogeneity compared to B30, which subsequently led to higher NO_x_ and lower smoke emissions, respectively. Also, oxygen content of methanol played an instrumental role behind increasing NO_x_ and lowering smoke emissions in case of M10 blend compared to B30 blend.
- A1 blend due to its higher CN compared to M10 blend caused more complete burning of pilot fuel and DP of main fuel was shortened effectively causing greater smoke emissions and lower NO_x_ emissions. Also, the overall combustion process shifted earlier toward TDC in case of A1 blend compared to B30 due to higher CN of the blend which further improved BSFC and BTE both.

Overall, it can be said that adding methanol and DTBP to base fuel can be an effective solution to the pertinent problem of greater smoke emissions associated with PI mode. The proposed strategy offers a minimal increase in smoke emissions under PI mode and maintains it nearly at the level of SI mode while maintaining all the benefits of PI mode.

The future work may include a proportional variation in methanol and DTBP into base fuel for analyzing its effect on engine variables under PI mode.

## Conflicts of Interest

The authors declare no conflicts of interest.

## Data Availability

The data that support the findings of this study are available from the corresponding author upon reasonable request.

## References

[1] L. Popoola , C. Nwogbu , U. Taura , Y. Asmara , A. Agbo , and P. Okonkwo , “Copper Corrosion in Blended Diesel‐Biodiesel: Corrosion Rate Evaluation and Characterization,” Chemical Engineering & Technology 48 (2025): 70053, 10.1002/ceat.70053.

[2] M. Elkelawy , A. Bastawissi , E. A. El Shenawy , et al., “Influence of Lean Premixed Ratio of PCCI‐DI Engine Fueled by Diesel/Biodiesel Blends on Combustion, Performance, and Emission Attributes; a Comparison Study,” Energy Conversion and Management: X 10 (2021): 100066, 10.1016/j.ecmx.2020.100066.

[3] S. Ahmed , T. Li , X. Y. Zhou , P. Yi , and R. Chen , “Quantifying the Environmental Footprints of Biofuels for Sustainable Passenger Ship Operations,” Renewable and Sustainable Energy Reviews 207 (2025): 114919, 10.1016/j.rser.2024.114919.

[4] M. Elkelawy , S. E. H. Etaiw , H. A. E. Bastawissi , et al., “Study of Diesel‐Biodiesel Blends Combustion and Emission Characteristics in a CI Engine by Adding Nanoparticles of Mn (II) Supramolecular Complex,” Atmospheric Pollution Research 11, no. 1 (2020): 117–128, 10.1016/j.apr.2019.09.021.

[5] V. Kumbhar , A. Pandey , C. R. Sonawane , A. S. El‐Shafay , H. Panchal , and A. J. Chamkha , “Statistical Analysis on Prediction of Biodiesel Properties from Its Fatty Acid Composition,” Case Studies in Thermal Engineering 30 (2022): 101775, 10.1016/j.csite.2022.101775.

[6] S. Torkzaban , M. Feyzi , and L. Norouzi , “A Novel Biodegradable Nanoparticle as a Robust Biodiesel Antioxidant, and Fuel Additive for Improvement of Diesel Engine Performance and Exhaust Emission,” Renewable Energy 242 (2025): 122345, 10.1016/j.renene.2025.122345.

[7] S. Boosala and B. Balakrishna , “Combustion, Emission & Performance Analysis of Surfactant Mixed‐MoO3 Nanoparticles Dispersed Gossypium Arboreum Biodiesel,” Energy Sources, Part A: Recovery, Utilization, and Environmental Effects 47 (2025): 3692–3711, 10.1080/15567036.2025.2454538.

[8] M. S. Chandra Sekar , V. R. Ananthan , N. Baskaran , H. K. Suresh Kumar , and R. Arumugam , “Combustion, Performance, and Emission Study on the Octanol‐ Neem Biodiesel Blends Fueled Diesel Engine,” Energy Sources, Part A: Recovery, Utilization, and Environmental Effects 46 (2024): 6167–6179, 10.1080/15567036.2020.1741736.

[9] F. Y. Hagos , O. M. Ali , R. Mamat , and A. A. Abdullah , “Effect of Emulsification and Blending on the Oxygenation and Substitution of Diesel Fuel for Compression Ignition Engine,” Renewable and Sustainable Energy Reviews 75 (2017): 1281–1294, 10.1016/j.rser.2016.11.113.

[10] S. Ma , Q. Guo , J. Wei , et al., “Analyzing the Effect of Carbon Nanoparticles on the Combustion Performance and Emissions of a DI Diesel Engine Fueled with the Diesel‐Methanol Blend,” Energy 300 (2024): 131616, 10.1016/j.energy.2024.131616.

[11] Z. Zhang , J. Tian , G. Xie , et al., “Investigation on the Combustion and Emission Characteristics of Diesel Engine Fueled with Diesel/Methanol/*n*‐butanol Blends,” Fuel 314 (2022): 123088, 10.1016/j.fuel.2021.123088.

[12] A. I. EL‐Seesy , M. S. Waly , Z. He , H. M. El‐Batsh , A. Nasser , and R. M. El‐Zoheiry , “Enhancement of the Combustion and Stability Aspects of Diesel‐Methanol‐Hydrous Methanol Blends Utilizing n‐octanol, Diethyl Ether, and Nanoparticle Additives,” Journal of Cleaner Production 371 (2022): 133673, 10.1016/j.jclepro.2022.133673.

[13] T. N. Verma , P. Nashine , P. K. Chaurasiya , et al., “The Effect of Ethanol‐Methanol‐Diesel‐Microalgae Blends on Performance, Combustion and Emissions of a Direct Injection Diesel Engine,” Sustainable Energy Technologies and Assessments 42 (2020): 100851, 10.1016/j.seta.2020.100851.

[14] P. Karvounis and G. Theotokatos , “Parametric Optimisation of Diesel–methanol Injection Timings of a Dual‐Fuel Marine Engine Operating with High Methanol Fraction Using CFD,” Applied Thermal Engineering 264 (2025): 125433, 10.1016/j.applthermaleng.2025.125433.

[15] H. Wei , C. Yao , W. Pan , et al., “To Meet Demand of Euro V Emission Legislation Urea Free for HD Diesel Engine with DMCC,” Fuel 207 (2017): 33–46, 10.1016/j.fuel.2017.06.070.

[16] I. Yusri , R. Mamat , G. Najafi , et al., “Alcohol Based Automotive Fuels from First Four Alcohol family in Compression and Spark Ignition Engine: a Review on Engine Performance and Exhaust Emissions,” Renewable and Sustainable Energy Reviews 77 (2017): 169–181, 10.1016/j.rser.2017.03.080.

[17] Y. Çelebi and H. Aydın , “An Overview on the Light Alcohol Fuels in Diesel Engines,” Fuel 236 (2019): 890–911, 10.1016/j.fuel.2018.08.138.

[18] E. W. De Menezes , R. Da Silva , R. Cataluña , and R. J. C. Ortega , “Effect of Ethers and Ether/Ethanol Additives on the Physicochemical Properties of Diesel Fuel and on Engine Tests,” Fuel 85 (2006): 815–822, 10.1016/j.fuel.2005.08.027.

[19] T. T. Truong , X. P. Nguyen , V. V. Pham , V. V. Le , A. T. Le , and V. T. Bui , “Effect of Alcohol Additives on Diesel Engine Performance: a Review,” Energy Sources, Part A: Recovery, Utilization, and Environmental Effects nd 47 (2025): 1–25, 10.1080/15567036.2021.2011490.

[20] K. Nanthagopal , R. S. Kishna , A. E. Atabani , A. H. Al‐Muhtaseb , G. Kumar , and B. Ashok , “A Compressive Review on the Effects of Alcohols and Nanoparticles as an Oxygenated Enhancer in Compression Ignition Engine,” Energy Conversion and Management 203 (2020): 112244, 10.1016/j.enconman.2019.112244.

[21] H. Chen , X. Su , J. He , and B. Xie , “Investigation on Combustion and Emission Characteristics of a Common Rail Diesel Engine Fueled with Diesel/n‐pentanol/Methanol Blends,” Energy 167 (2019): 297–311, 10.1016/j.energy.2018.10.199.

[22] H. Li , S. Xia , H. Luo , and P. Ma , “Experimental and Computational Study on the Compatibility of Biodiesel/Diesel/Methanol Blended Fuel,” Fuel 173 (2016): 52–59, 10.1016/j.fuel.2016.01.036.

[23] D. H. Qi , H. Chen , L. M. Geng , Y. Z. H. Bian , and X. C. H. Ren , “Performance and Combustion Characteristics of Biodiesel–diesel–methanol Blend Fuelled Engine,” Applied Energy 87 (2010): 1679–1686, 10.1016/j.apenergy.2009.10.016.

[24] M. Vargün , I. Turgut Yılmaz , and C. Sayın , “Investigation of Performance, Combustion and Emission Characteristics in a Diesel Engine Fueled with Methanol/Ethanol/nHeptane/Diesel Blends,” Energy 257 (2022): 124740, 10.1016/j.energy.2022.124740.

[25] J. Huang , H. Xiao , X. Yang , F. Guo , and X. Hu , “Effects of Methanol Blending on Combustion Characteristics and Various Emissions of a Diesel Engine Fueled with Soybean Biodiesel,” Fuel 282 (2020): 118734, 10.1016/j.fuel.2020.118734.

[26] Y. Jiao , R. Liu , Z. Zhang , et al., “Comparison of Combustion and Emission Characteristics of a Diesel Engine Fueled with Diesel and Methanol‐Fischer‐Tropsch Diesel‐Biodiesel‐Diesel Blends at Various Altitudes,” Fuel 243 (2019): 52–59, 10.1016/j.fuel.2019.01.107.

[27] X. Li , H. Gao , L. Zhao , Z. Zhang , X. He , and F. Liu , “Combustion and Emission Performance of a Split Injection Diesel Engine in a Double Swirl Combustion System,” Energy 114 (2016): 1135–1146, 10.1016/j.energy.2016.08.092.

[28] H. Dave , D. Solanki , and P. Naik , “Effect of Pilot Fuel Quantity and Fuel Injection Pressure on Combustion, Performance and Emission Characteristics of an Automotive Diesel Engine,” International Journal of Thermofluids 21 (2024): 100570, 10.1016/j.ijft.2024.100570.

[29] K. Lu , H. Qiu , Z. Chen , L. Shi , and K. Deng , “Environmental Adaptability Method for Improving the Cold Start Performance of the Diesel Engine Based on Pilot Injection Strategy,” Energy 281 (2023): 128215, 10.1016/j.energy.2023.128215.

[30] K. Subramanian , S. A. Paramasivam , D. Dillikannan , and R. Jayabal , “Effect of Pilot Fuel Injection Strategies and EGR on a CRDI Diesel Engine Powered by Simmondsia Chinensis Seed Biodiesel‐Methyl Acetate Blend,” Sustainable Energy Technologies and Assessments 58 (2023): 103345, 10.1016/j.seta.2023.103345.

[31] R. Huang , X. Guo , H. Huang , M. Pan , T. Wang , and H. Lei , “Assessment of Pilot Injection Strategies and n‐pentanol Additive Effects on Engine Performance and Emissions,” Fuel 257 (2019): 115999, 10.1016/j.fuel.2019.115999.

[32] J. Jeon and S. Park , “Effects of Pilot Injection Strategies on the Flame Temperature and Soot Distributions in an Optical CI Engine Fueled with Biodiesel and Conventional Diesel,” Applied Energy 160 (2015): 581–591, 10.1016/j.apenergy.2015.09.075.

[33] Z. Zheng , L. Yue , H. Liu , Y. Zhu , X. Zhong , and M. Yao , “Effect of Two‐Stage Injection on Combustion and Emissions under High EGR Rate on a Diesel Engine by Fueling Blends of Diesel/Gasoline, Diesel/n‐butanol, Diesel/Gasoline/n‐butanol and Pure Diesel,” Energy Conversion and Management 90 (2015): 1–11, 10.1016/j.enconman.2014.11.011.

[34] H. Dave , H. Choksi , M. I. H. Siddiqui , S. Dixit , and A. Markiewicz , “Investigation of Diesel Engine Characteristics under Pilot Injection Mode Using Diesel‐DTBP Blends,” Scientific Reports 15 (2025): 22988, 10.1038/s41598-025-05687-6. 40596189 PMC12218341

[35] P. Carlucci , A. Ficarella , and D. Laforgia , “Effects of Pilot Injection Parameters on Combustion for Common Rail Diesel Engines,” SAE Transactions 112 (2003): 932–943, 10.4271/2003-01-0700.

[36] İ. Örs , “Experimental Investigation of the Cetane Improver and Bioethanol Addition for the Use of Waste Cooking Oil Biodiesel as an Alternative Fuel in Diesel Engines,” Journal of the Brazilian Society of Mechanical Sciences and Engineering 42 (2020): 177, 10.1007/s40430-020-2270-1.

[37] H. Imdadul , H. Masjuki , M. Kalam , et al., “Evaluation of Oxygenated *n*‐butanol‐Biodiesel Blends Along with Ethyl Hexyl Nitrate as Cetane Improver on Diesel Engine Attributes,” Journal of Cleaner Production 141 (2017): 928–939, 10.1016/j.jclepro.2016.09.140.

[38] E. Ileri , “Experimental Study of 2‐ethylhexyl Nitrate Effects on Engine Performance and Exhaust Emissions of a Diesel Engine Fueled with n‐butanol or 1‐pentanol Diesel–sunflower Oil Blends,” Energy Conversion and Management 118 (2016): 320–330, 10.1016/j.enconman.2016.04.015.

[39] S. Mishra , A. Chauhan , and K. B. Mishra , “Role of Binary and Ternary Blends of WCO Biodiesel on Emission Reduction in Diesel Engine,” Fuel 262 (2020): 116604, 10.1016/j.fuel.2019.116604.

[40] P. Paneerselvam , G. Venkadesan , M. S. Panithasan , G. Alaganathan , S. Wierzbicki , and M. Mikulski , “Evaluating the Influence of Cetane Improver Additives on the Outcomes of a Diesel Engine Characteristics Fueled with Peppermint Oil Diesel Blend,” Energies 14 (2021): 2786, 10.3390/en14102786.

[41] Y. Devarajan , D. Munuswamy , B. Nagappan , and S. Ganesan , “Detailed Study on the Effect of Different Ignition Enhancers in the Binary Blends of Diesel/Biodiesel as a Possible Substitute for Unaltered Compression Ignition Engine,” Petroleum Science 17 (2020): 1151–1158, 10.1007/s12182-020-00463-9.

[42] M. M. Musthafa , “Development of Performance and Emission Characteristics on Coated Diesel Engine Fuelled by Biodiesel with Cetane Number Enhancing Additive,” Energy 134 (2017): 234–239, 10.1016/j.energy.2017.06.012.

[43] H. Huang , Q. Liu , R. Yang , T. Zhu , R. Zhao , and Y. Wang , “Investigation on the Effects of Pilot Injection on Low Temperature Combustion in High‐Speed Diesel Engine Fueled with n ‐butanol–Diesel Blends,” Energy Conversion and Management 106 (2015): 748–758, 10.1016/j.enconman.2015.10.031.

[44] J. Wei , Z. Yin , C. Wang , et al., “Impact of Aluminium Oxide Nanoparticles as an Additive in Diesel‐Methanol Blends on a Modern DI Diesel Engine,” Applied Thermal Engineering 185 (2021): 116372, 10.1016/j.applthermaleng.2020.116372.

[45] Z. H. Huang , H. B. Lu , D. M. Jiang , et al., “Engine Performance and Emissions of a Compression Ignition Engine Operating on the Diesel‐Methanol Blends,” Proceedings of the Institution of Mechanical Engineers, Part D: Journal of Automobile Engineering 218 (2004): 435–447, 10.1243/095440704773599944.

[46] Q. Chen , C. Wang , K. Shao , Y. Liu , X. Chen , and Y. Qian , “Analyzing the Combustion and Emissions of a DI Diesel Engine Powered by Primary Alcohol (methanol, ethanol, *n*‐butanol)/Diesel Blend with Aluminum Nano‐Additives,” Fuel 328 (2022): 125222, 10.1016/j.fuel.2022.125222.

